# Gastrointestinal toxicity of targeted cancer therapies in the United States: Clinicopathologic patterns, FDA safety frameworks, and implications for national patient protection

**DOI:** 10.18632/oncoscience.643

**Published:** 2026-02-06

**Authors:** Muhammad Moseeb Ali Hashim, Muhammad Ahsan, Muhammad Aizaz Mohsin Khan, Hafsa Hameed Thakur, Talha Kamran Khan, Kamran Zahoor, Sania Muzaffar, Feroza Fatima, Shamama Tu Zahra, Ammara Naeem, Mahima Gandhi, Muhammad Usama Ashraf, Pir Maroof Qureshi

**Affiliations:** ^1^Department of Pathology and Laboratory Sciences, University of Missouri-Columbia, Missouri, MO 65201, USA; ^2^Department of Histopathology, Chughtai Institute of Pathology, Lahore, Punjab, Pakistan; ^3^School of Medicine, University of Buckingham, Buckingham, UK; ^4^Alpha Clinical Developments Limited, Salford M3 7NA, UK; ^5^Pathology and Laboratory Medicine, University of California Davis Health, California, CA 95817, USA; ^6^Department of Neurology, University of Missouri-Columbia, Missouri, MO 65201, USA; ^7^Department of Pathology, Dow University of Health Sciences, Karachi 74200, Pakistan; ^8^Department of Pathology, Primary Health Care Corporation, Doha, Qatar; ^9^Department of Pathology, Lahore General Hospital, Lahore 54000, Pakistan; ^10^Department of Medicine, Pakistan Kidney Patient’s Association, Islamabad, Pakistan; ^11^Department of Pathology, Dr Ziauddin Hospital Karachi, Karachi 74700, Pakistan; ^12^Excellent Medical Associates, Chicago, IL 60462, USA; ^13^Department of Pathology, Liaquat University hospital, Hyderabad 71000, Pakistan

**Keywords:** gastrointestinal toxicity, tyrosine kinase inhibitors, antibody-drug conjugates, CAR-T cell therapy, targeted cancer therapy

## Abstract

Background: As precision oncology advances, non-immune checkpoint targeted therapies such as tyrosine kinase inhibitors (TKIs), antibody-drug conjugates (ADCs), and chimeric antigen receptor T-cell (CAR-T) therapies are increasingly used across gastrointestinal (GI) and non-GI malignancies. While these agents have transformed cancer treatment, they are also associated with a broad spectrum of GI toxicities that remain underrecognized in both clinical practice and pathology.

Objective: This review comprehensively examines the mechanisms, clinicopathological features, and management strategies of GI toxicity induced by TKIs, ADCs, and CAR-T therapies, emphasizing the diagnostic role of pathologists in identifying treatment-related injury patterns.

Methods: We synthesized data from pivotal clinical trials, FDA drug labeling, post-marketing surveillance (FAERS), and real-world histopathologic descriptions of GI adverse events. SEER data on GI malignancies treated with targeted therapies were also reviewed to highlight epidemiologic context.

Results: TKIs may induce mucosal ischemia, apoptosis, or colitis-like inflammation due to angiogenesis inhibition and off-target effects. ADCs contribute to epithelial injury through cytotoxic payloads, while CAR-T therapy is associated with cytokine-mediated GI inflammation. Histological findings range from apoptotic enteropathy to ulcerative colitis and mimic infections, GVHD, or autoimmune disease. Misdiagnosis can lead to treatment delays or unnecessary dose reductions.

Conclusions: The landscape of GI toxicity from targeted cancer therapies is expanding rapidly. Accurate recognition of characteristic pathology patterns and integration with clinical history are crucial for safe and effective management. Enhanced pharmacovigilance, pathology-oncology collaboration, and incorporation of national surveillance data (FAERS, SEER) are essential to advancing precision medicine and patient safety.

## INTRODUCTION

The landscape of modern oncology has evolved dramatically over the past two decades, propelled by the development and widespread use of molecularly targeted agents and cell-based immunotherapies. Among these, tyrosine kinase inhibitors (TKIs), antibody–drug conjugates (ADCs), and chimeric antigen receptor T-cell (CAR-T) therapies have emerged as pivotal therapeutic modalities across a wide spectrum of malignancies, including gastrointestinal (GI) cancers such as gastrointestinal stromal tumors (GISTs), colorectal carcinoma, pancreatic ductal adenocarcinoma, and HER2-positive gastric cancer [1–4]. These therapies offer the advantage of specificity, often achieving significant improvements in survival, disease control, and quality of life.

However, alongside these therapeutic advances, a growing body of evidence has highlighted the potential for these agents to cause a variety of off-target toxicities, particularly within the gastrointestinal tract. Unlike traditional cytotoxic chemotherapy, the GI toxicities associated with targeted therapies are often idiosyncratic, variable in onset and severity, and mechanistically distinct across drug classes. They frequently present with histologic and clinical features that overlap with other disease processes, such as infection, inflammatory bowel disease (IBD), ischemia, and graft-versus-host disease (GVHD), leading to diagnostic ambiguity and delays in appropriate management [5, 6].

Tyrosine kinase inhibitors, such as regorafenib, lenvatinib, and sunitinib, have been associated with mucosal injury, diarrhea, and ischemic-type colitis through their antiangiogenic and multikinase inhibitory effects. ADCs like trastuzumab deruxtecan and inotuzumab ozogamicin are conjugated with potent cytotoxic payloads, which may cause mucositis, colitis, and gastrointestinal hemorrhage secondary to direct epithelial injury or bystander effects [7, 8]. CAR-T therapies, although primarily used for hematologic malignancies, have been linked to cytokine-mediated GI inflammation, enteritis, and even colitis, particularly in the setting of cytokine release syndrome (CRS) and immune effector cell–associated neurotoxicity syndrome (ICANS) [9–11].

Despite the increasing prevalence of these toxicities, current literature remains limited in its coverage of the histopathologic features and clinicopathologic correlations of GI injury secondary to non–immune checkpoint targeted therapies. Pathologists often face challenges in identifying characteristic patterns of injury, especially when clinical histories are incomplete or when biopsy findings mimic other entities. The lack of standardized descriptors or classification systems for these toxicities further complicates pathology reporting and interdisciplinary communication.

Data derived from regulatory and public health sources such as the U.S. Food and Drug Administration (FDA) and the FDA Adverse Event Reporting System (FAERS) reinforce the clinical relevance of this issue. Post-marketing surveillance data have documented thousands of GI adverse events associated with kinase inhibitors and ADCs, yet these findings remain under-integrated into routine practice and academic discourse. Concurrently, epidemiologic datasets like the Surveillance, Epidemiology, and End Results (SEER) program show rising incidence rates of GI malignancies for which these therapies are indicated, underscoring the importance of understanding their toxicity profiles in a population-level context [12–14].

This review aims to provide a comprehensive and pathology-focused synthesis of the gastrointestinal toxicities associated with TKIs, ADCs, and CAR-T cell therapies. By exploring the mechanistic pathways, histopathologic features, regulatory surveillance data, and clinical implications, we seek to empower pathologists, oncologists, and multidisciplinary teams with a practical reference to recognize, report, and manage these emerging toxicities. Ultimately, improved awareness and timely diagnosis will support safer treatment delivery, more effective pharmacovigilance, and higher-quality cancer care.

## OVERVIEW OF NON–IMMUNE CHECKPOINT TARGETED THERAPIES IN GI ONCOLOGY

### Tyrosine kinase inhibitors (TKIs)

Tyrosine kinase inhibitors are among the most established classes of targeted agents in gastrointestinal oncology. They act by blocking intracellular signaling pathways critical for tumor growth, angiogenesis, and metastasis. In gastrointestinal stromal tumors (GISTs), the discovery of activating mutations in KIT and PDGFRA led to the approval of imatinib, which demonstrated unprecedented response rates in advanced disease [15, 16]. However, resistance is common, necessitating subsequent agents such as sunitinib, regorafenib, and ripretinib [17, 18]. TKIs also play a role in hepatocellular carcinoma (HCC), where sorafenib was the first systemic therapy to improve survival, followed by lenvatinib as an alternative first-line option [19, 20]. These drugs inhibit vascular endothelial growth factor receptors (VEGFR) in addition to other kinases, accounting for both their efficacy and gastrointestinal toxicity, which includes diarrhea, mucosal ulceration, ischemic changes, and bleeding [21].

### Antibody–drug conjugates (ADCs)

ADCs represent a rapidly expanding category of therapies that link monoclonal antibodies with potent cytotoxic payloads. In GI oncology, trastuzumab deruxtecan (DS-8201a) has emerged as a breakthrough therapy for HER2-positive gastric and gastroesophageal junction cancers after progression on trastuzumab [22]. Clinical trials demonstrated durable responses, though at the cost of significant gastrointestinal side effects including nausea, vomiting, diarrhea, and mucositis [23, 24]. The mechanism of toxicity may involve both on-target damage to normal HER2-expressing epithelial cells and off-target bystander effects from payload release [25]. Other ADCs, such as sacituzumab govitecan (anti–Trop-2) and enfortumab vedotin (anti–Nectin-4), while not yet approved for GI cancers, are under investigation in colorectal and gastric cancer with promising preliminary results [26, 27].

### Chimeric antigen receptor T-Cell (CAR-T) therapy

CAR-T cell therapy has transformed the treatment of hematologic malignancies and is now being explored for gastrointestinal cancers. Early-phase trials have targeted antigens such as carcinoembryonic antigen (CEA), claudin 18.2, and glypican-3 in gastric, pancreatic, and hepatocellular carcinomas [28]. While clinical efficacy in solid tumors remains modest, CAR-T therapy is associated with distinct toxicities, including cytokine release syndrome (CRS) and immune effector cell–associated neurotoxicity syndrome (ICANS). These systemic inflammatory responses frequently involve the gastrointestinal tract, producing colitis, enteritis, and, in rare cases, gastrointestinal bleeding [29]. Histologically, these lesions can mimic inflammatory bowel disease or ischemic colitis, underscoring the role of pathology in differential diagnosis [30].

### Bispecific antibodies

Bispecific antibodies, particularly CD3-directed T-cell engagers such as teclistamab, epcoritamab, and blinatumomab, represent an emerging class of non–checkpoint immunotherapies with expanding use across hematologic and select solid malignancies [31]. These agents bind CD3 on T cells and a tumor-associated antigen (e.g., BCMA, CD20, HER2), inducing potent T-cell activation and targeted cytotoxicity. Similar to CAR-T therapies, this immune synapse formation triggers cytokine release, most commonly IL-6 and IL-1β, which can manifest clinically as CRS-associated diarrhea and systemic inflammation [32]. Gastrointestinal toxicities include diarrhea, immune-mediated enterocolitis (uncommon but documented), and mucosal apoptosis during high-grade cytokine activation. Unlike CAR-T–associated GI injury, which often appears during or after CRS escalation, bispecific antibody–related GI events tend to occur earlier in the step-up dosing phase, reflecting their rapid and repeat T-cell stimulation kinetics [33].

### Other targeted therapies of relevance

In addition to TKIs, ADCs, and CAR-T, other targeted therapies contribute to gastrointestinal oncology. The mammalian target of rapamycin (mTOR) inhibitor everolimus is approved for pancreatic neuroendocrine tumors and has shown activity in HCC, with gastrointestinal side effects such as stomatitis, diarrhea, and enteritis [34, 35]. Poly (ADP-ribose) polymerase (PARP) inhibitors, including olaparib and rucaparib, though more established in ovarian and breast cancers, have shown activity in GI cancers harboring BRCA mutations or homologous recombination deficiency, sometimes producing gastrointestinal toxicities such as nausea, vomiting, and diarrhea [36, 37]. Bispecific T-cell engagers (BiTEs) and inhibitors of epigenetic regulators are in early development for GI malignancies and will likely expand the spectrum of potential gastrointestinal adverse events in the near future [38]. Targeted cancer therapies and associated GI toxicities are summarized in Table 1.

**Table 1:** Targeted cancer therapies in gastrointestinal oncology and associated GI toxicities

Drug class	Representative agents	Approved indications (GI Focus)	Common GI toxicities
**Tyrosine Kinase Inhibitors (TKIs)**	Imatinib, Sunitinib, Regorafenib, Ripretinib, Sorafenib, Lenvatinib, Cabozantinib	GIST (KIT/PDGFRA mutations), Hepatocellular carcinoma, Pancreatic neuroendocrine tumors, Biliary tract cancers	Diarrhea, nausea, vomiting, mucosal ulceration, ischemic colitis, GI bleeding
**Antibody–Drug Conjugates (ADCs)**	Trastuzumab deruxtecan, Sacituzumab govitecan, Enfortumab vedotin	HER2-positive gastric and gastroesophageal junction cancer; *under investigation for colorectal and gastric cancer*	Nausea, diarrhea, mucositis, colitis, GI hemorrhage
**CAR-T Cell Therapies**	Axicabtagene ciloleucel, Tisagenlecleucel, Claudin18.2-CAR-T, CEA-CAR-T	FDA-approved for hematologic malignancies; early-phase trials in gastric, pancreatic, and hepatocellular carcinoma	Cytokine-mediated colitis, enteritis, diarrhea, GI bleeding
**mTOR Inhibitors**	Everolimus	Pancreatic neuroendocrine tumors, Hepatocellular carcinoma	Stomatitis, diarrhea, enteritis, mucosal inflammation
**PARP Inhibitors**	Olaparib, Rucaparib	GI cancers with BRCA mutations or homologous recombination deficiency (investigational)	Nausea, vomiting, diarrhea, mucositis

Table 1 summarizes selected FDA-approved and investigational non–immune checkpoint targeted therapies relevant to gastrointestinal oncology. Representative agents are listed under each drug class, with an emphasis on those with either FDA approval for GI cancers or ongoing clinical investigation. Gastrointestinal toxicities include both clinical adverse events and histopathologically confirmed lesions reported in trials, FDA labeling, and case series. Abbreviations: ADC: Antibody-Drug Conjugate; BiTE: Bispecific T-cell Engager; BRCA: Breast Cancer gene (BRCA1/2 mutations associated with homologous recombination deficiency); CAR-T: Chimeric Antigen Receptor T-cell therapy; CEA: Carcinoembryonic Antigen; GIST: Gastrointestinal Stromal Tumor; GI: Gastrointestinal; HDAC: Histone Deacetylase; mTOR: Mammalian Target of Rapamycin; PARP: Poly (ADP-ribose) Polymerase; PDGFRA: Platelet-Derived Growth Factor Receptor Alpha; TKI: Tyrosine Kinase Inhibitor; VEGFR: Vascular Endothelial Growth Factor Receptor.

## MECHANISMS OF GASTROINTESTINAL TOXICITY

The gastrointestinal adverse effects of targeted cancer therapies arise from complex interactions between pharmacologic targets, host tissue biology, and systemic immune responses. Unlike cytotoxic chemotherapy, which causes predictable mucosal injury through cell-cycle inhibition, targeted therapies produce heterogeneous patterns of toxicity that reflect both on-target and off-target mechanisms. Understanding these mechanisms is essential for interpreting histopathologic findings and guiding patient management.

### Direct epithelial injury and disruption of stem cell niche

Several targeted agents exert direct cytotoxic effects on gastrointestinal epithelial cells. Antibody–drug conjugates (ADCs) are the prototypical example, as their payloads—typically topoisomerase inhibitors or microtubule-disrupting agents can be released within normal mucosal cells or diffuse into surrounding tissues. Trastuzumab deruxtecan, for example, has been associated with mucositis, diarrhea, and colitis due to bystander epithelial damage in HER2-expressing mucosa [7]. Similarly, poly (ADP-ribose) polymerase (PARP) inhibitors induce DNA repair defects that sensitize rapidly dividing gastrointestinal epithelial cells to injury, leading to diarrhea and mucosal inflammation [39].

Targeted therapies may also disrupt the intestinal epithelial stem cell niche, an emerging and increasingly recognized mechanism of injury. The regenerative capacity of the gastrointestinal mucosa depends on LGR5^+^ intestinal stem cells residing at the crypt base, supported by a tightly regulated microenvironment shaped by Wnt, Notch, EGFR, and mTOR signaling [40]. Several targeted agents particularly TKIs, mTOR inhibitors, and select ADCs can perturb these pathways, leading to impaired stem cell renewal, delayed crypt regeneration, and heightened vulnerability to mucosal stress. Disruption of the niche may manifest histologically as crypt dropout, blunted regenerative response, or increased apoptosis, contributing to prolonged diarrhea, mucosal atrophy, and delayed healing even after drug discontinuation [41, 42]. This evolving concept underscores how targeted therapy–induced epithelial injury extends beyond direct cytotoxicity to include dysregulation of epithelial homeostasis and repair.

### Anti-angiogenic and ischemic mechanisms

Tyrosine kinase inhibitors (TKIs) with anti-VEGFR activity can cause gastrointestinal ischemia and ulceration by disrupting vascular integrity. Sorafenib, regorafenib, and lenvatinib are known to reduce microvascular density in the intestinal mucosa, predisposing to ischemic-type colitis and gastrointestinal bleeding [43]. Histologically, these lesions often display mucosal necrosis, thrombosed capillaries, and lamina propria hyalinization, mimicking ischemic colitis from vascular disease. Such vascular-mediated injury distinguishes VEGF-targeted TKIs from other classes of targeted therapies.

### Apoptotic enteropathy and crypt injury

Agents that modulate downstream signaling pathways, including mTOR inhibitors such as everolimus, induce characteristic patterns of apoptotic injury in the gastrointestinal tract. These lesions may resemble graft-versus-host disease (GVHD) or immune checkpoint inhibitor–related enterocolitis, with apoptotic bodies in crypt bases and focal crypt dropout [44]. Recognition of this mechanism is essential to avoid misdiagnosis, as the clinical management differs significantly from GVHD or infectious etiologies.

### Immune-mediated and cytokine-driven toxicity

CAR-T cell therapy represents a distinct paradigm of gastrointestinal toxicity mediated by immune hyperactivation rather than direct tissue targeting. Cytokine release syndrome (CRS) produces high systemic levels of interleukin-6, interferon-γ, and tumor necrosis factor-α, which can disrupt mucosal immune homeostasis and lead to colitis or enteritis [11]. Pathology specimens often reveal mixed inflammatory infiltrates, crypt apoptosis, and mucosal ulceration, sometimes indistinguishable from autoimmune colitis [45, 46]. The overlap with infectious and inflammatory conditions necessitates careful clinicopathologic correlation.

### Microbiome dysregulation

Emerging data suggest that targeted therapies can alter the gut microbiome, which may contribute to gastrointestinal toxicity. TKIs such as sunitinib and cabozantinib have been shown to disrupt microbial diversity, increasing the abundance of pro-inflammatory taxa [5]. These alterations may potentiate mucosal immune activation, lower the threshold for diarrhea, and exacerbate colitis. Although still under active investigation, microbiome disruption may partially explain interpatient variability in toxicity profiles [47, 48].

### Off-target kinase inhibition

**Figure 1:**
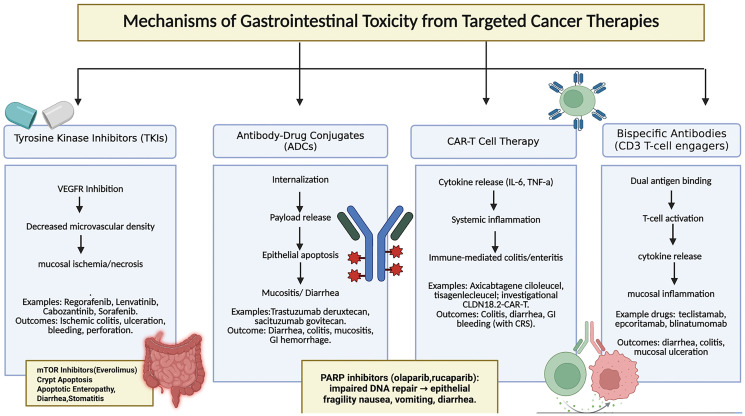
Mechanisms of Gastrointestinal Toxicity from Targeted Cancer Therapies. This schematic illustrates the major mechanistic pathways by which targeted therapies cause gastrointestinal toxicity. TKIs primarily act through vascular compromise, ADCs through direct epithelial injury, and CAR-T cells via cytokine-mediated inflammation. Other classes, including mTOR and PARP inhibitors, contribute apoptotic and DNA-repair–linked injury patterns. Abbreviations: ADC: Antibody–Drug Conjugate; CAR-T: Chimeric Antigen Receptor T-cell; CLDN18.2: Claudin 18.2; CRS: Cytokine Release Syndrome; GI: Gastrointestinal; mTOR: Mammalian Target of Rapamycin; PARP: Poly (ADP-ribose) Polymerase; TKI: Tyrosine Kinase Inhibitor; VEGF/VEGFR: Vascular Endothelial Growth Factor/(Receptor).

TKIs frequently inhibit multiple kinases beyond their intended oncogenic target, and this polypharmacology accounts for a broad range of off-target gastrointestinal effects. Inhibition of platelet-derived growth factor receptor (PDGFR) and c-KIT in normal intestinal cells contributes to impaired mucosal repair, while off-target inhibition of epidermal growth factor receptor (EGFR) can promote diarrhea and epithelial atrophy [49]. Recognition of these off-target effects helps explain why toxicity profiles vary substantially between TKIs despite shared primary targets. Mechanisms of gastrointestinal toxicity from target cancer therapies is illustrated in Figure 1.

## CLINICAL SPECTRUM AND SITES OF GASTROINTESTINAL TOXICITY

The clinical presentation of targeted-therapy–related gastrointestinal (GI) injury spans the entire alimentary tract and is shaped by drug class, cumulative dose, concomitant medications, and baseline mucosal vulnerability. Time to onset is variable ranging from days for cytotoxic-payload–driven mucositis to weeks for anti-angiogenic ischemic patterns and histology often overlaps with infection, inflammatory bowel disease, ischemia, or immune-mediated injury. Close clinicopathologic correlation, awareness of class-specific signatures, and knowledge of regulatory safety data are essential to avoid misdiagnosis and unnecessary treatment interruption.

### Upper GI tract (esophagus and stomach)

Upper-tract toxicity most often reflects epithelial-injury and anti-angiogenic effects. Antibody–drug conjugates (ADCs) such as trastuzumab deruxtecan produce prominent nausea, vomiting, stomatitis/mucositis, and dyspepsia; endoscopy may show erythematous or erosive gastritis, and biopsies demonstrate surface erosion with apoptotic epithelial damage attributable to topoisomerase-I payload bystander effect [50]. VEGF-pathway inhibition (e.g., with lenvatinib or sorafenib) is associated with hemorrhagic gastritis and mucosal ulceration, consistent with impaired mucosal vascular integrity and repair [51, 52]. Clinically significant upper-GI bleeding and ulceration are listed in prescribing information across anti-angiogenic agents and should prompt evaluation for concurrent NSAID/anticoagulant use and *Helicobacter pylori* eradication where relevant [53].

### Small intestine

Small-bowel involvement ranges from secretory diarrhea and crampy abdominal pain to ischemic enteritis and, rarely, perforation. Anti-VEGF antibodies and multi-kinase VEGFR inhibitors increase the risk of GI perforation and ischemic-type injury, likely via microvascular compromise; histology shows mucosal necrosis, withered crypts, lamina propria hyalinization, and capillary thrombosis [51, 54]. ADCs and PARP inhibitors can cause drug-induced enteritis characterized by epithelial apoptosis and brisk mitotic arrest without a dominant neutrophilic component [54, 55]. In practice, exclusion of infectious etiologies (especially *Clostridioides difficile*) and careful medication reconciliation are crucial when biopsies show nonspecific active enteritis in patients on these agents.

### Colon and rectum

The colon is the most frequently biopsied site and exhibits several recurring patterns. VEGFR-targeting TKIs (e.g., regorafenib, lenvatinib, cabozantinib) produce ischemic-type colitis or ulceration with segmental distribution, hemorrhage, and endoscopic dusky mucosa; biopsies reveal ischemic injury with superficial necrosis and lamina propria hemorrhage [56]. mTOR inhibition (everolimus) and cytotoxic-payload ADCs may cause an apoptotic colitis/enteropathy pattern with increased crypt-base apoptosis and focal crypt dropout that can mimic graft-versus-host disease or checkpoint-inhibitor colitis; recognition of prominent apoptosis with relatively scant chronic architectural distortion supports a drug effect [57]. Irinotecan-analog payloads (e.g., sacituzumab govitecan) are linked to severe diarrhea and colitis; supportive care and, when indicated, antidiarrheals are standard while invasive infection is excluded [58]. CAR-T–associated systemic inflammation can manifest as colitis with mixed inflammatory infiltrates, crypt apoptosis, and erosions, often temporally related to cytokine release syndrome; pathologic features overlap with autoimmune colitis, necessitating integration of treatment timeline and biomarkers [59].

### Pancreas

Pancreatic toxicity is uncommon but reported with selected TKIs and mTOR inhibitors. Clinical presentations include asymptomatic enzyme elevations to acute pancreatitis. When pancreatitis occurs, it is typically early, reversible with drug interruption, and confounded by gallstone disease or hypertriglyceridemia; imaging may be normal or show interstitial edema. Package inserts for sunitinib and sorafenib acknowledge pancreatitis as a post-marketing event; management is supportive with temporary cessation and rechallenge only after resolution and exclusion of alternative causes [60, 61]. Everolimus more commonly causes stomatitis and hyperglycemia; pancreatitis is rare and diagnosis requires standard clinical criteria [62].

### Hepatic and biliary tract

Targeted agents frequently produce hepatobiliary adverse events that intersect with GI care. Multi-kinase inhibitors such as regorafenib carry a boxed warning for severe drug-induced liver injury; patterns include hepatocellular transaminitis with possible jaundice, typically within the first two months of therapy, warranting close laboratory monitoring and prompt dose modification per label guidance [63]. PI3Kδ inhibition with idelalisib is associated with high-grade transaminase elevations and, rarely, cholestatic injury; risk is greatest in the initial 12 weeks and improves with interruption or dose reduction [64]. Inotuzumab ozogamicin (anti-CD22 ADC) confers a clinically meaningful risk of hepatic sinusoidal obstruction syndrome/veno-occlusive disease, particularly around hematopoietic stem-cell transplantation; pathogenesis reflects endothelial-sinusoidal injury from the calicheamicin payload, and prevention centers on exposure minimization and transplant-timing strategies [65, 66]. Bile-duct–centered injury and drug-induced cholangitis are uncommon but reported across targeted classes; careful exclusion of obstruction and infectious cholangitis is essential before attributing causality.

## HISTOPATHOLOGICAL PATTERNS AND DIAGNOSTIC PITFALLS

The histopathologic spectrum of gastrointestinal injury caused by targeted therapies is remarkably diverse, reflecting the distinct pharmacologic mechanisms of these agents. Because many of these patterns overlap with infectious, autoimmune, and ischemic processes, awareness of characteristic morphologic features and integration with treatment history are essential to avoid misdiagnosis.

### Apoptotic enteropathy

Apoptotic injury is a recurring feature of gastrointestinal toxicity from mTOR inhibitors and several ADCs. Biopsies demonstrate increased apoptotic bodies in crypt bases, sometimes with crypt dropout and mucosal thinning. This pattern can closely mimic graft-versus-host disease (GVHD) or immune checkpoint inhibitor (ICI) colitis. Clinical context and lack of architectural distortion or chronic inflammatory infiltrates favor a drug-induced etiology [67].

### Ischemic-type injury

VEGFR-targeted TKIs frequently produce ischemic-type mucosal damage. Histology reveals surface necrosis, withered crypts, lamina propria hemorrhage, hyalinization, and thrombotic microangiopathy of mucosal capillaries. Unlike vascular occlusion from atherosclerotic disease, these changes are diffuse, often segmental, and temporally associated with TKI therapy. Differentiation from primary ischemic colitis is critical, as continued drug exposure risks severe perforation or hemorrhage [68, 69].

### Ulcerative and erosive colitis

Agents with cytotoxic payloads (e.g., sacituzumab govitecan, trastuzumab deruxtecan) often cause ulceration and mucosal erosions with mixed inflammatory infiltrates. Histology may show neutrophil-rich exudates and fibrinopurulent debris, resembling infectious colitis. Microbiologic testing is essential to rule out *Clostridioides difficile* or viral pathogens. In drug-induced settings, chronic architectural distortion is absent, helping to distinguish these lesions from idiopathic inflammatory bowel disease [70].

### Mixed inflammatory and autoimmune-like patterns

CAR-T–associated gastrointestinal toxicity is driven by systemic cytokine release and may manifest as mixed inflammatory infiltrates with crypt apoptosis, erosions, and ulceration. Pathologic features overlap substantially with autoimmune colitis and checkpoint inhibitor toxicity. In such cases, correlation with treatment timeline, cytokine release syndrome, and systemic biomarkers (e.g., elevated IL-6) supports the diagnosis [71].

### Hepatobiliary lesions

Targeted therapies also produce distinct hepatobiliary histopathology. Regorafenib and other TKIs can induce hepatocellular injury with lobular necrosis and cholestasis, while inotuzumab ozogamicin may cause hepatic sinusoidal obstruction syndrome (SOS), showing sinusoidal dilatation, centrilobular congestion, and perisinusoidal fibrosis [65]. Accurate recognition of these lesions is essential to prevent progression to fulminant hepatic failure, particularly in patients undergoing hematopoietic stem cell transplantation.

### Diagnostic pitfalls

The principal diagnostic challenge lies in differentiating drug-induced injury from mimics such as GVHD, ICI colitis, infection, and ischemia. Overinterpretation of apoptosis as GVHD can lead to unnecessary immunosuppression, while misattribution of ischemic injury to vascular occlusion may delay discontinuation of the offending drug. Close communication between oncologists, gastroenterologists, and pathologists, along with careful review of therapy timelines, remains the cornerstone of accurate diagnosis.

## FAERS AND FDA-LABEL EVIDENCE ON GI ADVERSE EVENTS

A rigorous understanding of gastrointestinal (GI) toxicity from targeted, non-ICI therapies requires triangulating two complementary sources: the FDA Adverse Event Reporting System (FAERS), which offers nationwide, post-marketing signal surveillance, and FDA-approved prescribing information, which provides trial-anchored incidence and severity. FAERS enables hypothesis generation but is not designed to prove causality or provide incidence; FDA labels enumerate event rates under defined study conditions but can under-represent rare or delayed toxicities. Judicious synthesis of both is therefore essential for patient safety in U.S. oncology practice [72].

### What FAERS can (and cannot) tell us

FAERS aggregates spontaneous reports from clinicians, manufacturers, and patients across the United States and is accessible through an interactive public dashboard. Signals of disproportionate reporting for preferred terms such as “diarrhea,” “colitis,” “intestinal perforation,” “GI hemorrhage,” and “stomatitis” can highlight drug–event pairs that warrant closer review (e.g., anti-VEGF–associated perforation, ADC-associated diarrhea/colitis). However, FAERS lacks a reliable denominator, is subject to duplicate and stimulated reporting, and cannot establish causality; signals must be validated against clinical and trial data before altering care [72].

Recent pharmacovigilance analyses using FAERS underscore class-level concerns for antibody-drug conjugates (ADCs), where GI adverse events—particularly diarrhea and colitis—appear over-represented relative to background expectations, supporting heightened monitoring and early supportive care pathways. While such studies help prioritize surveillance, their findings remain hypothesis-generating and should be interpreted alongside label and clinical-trial evidence [73].

### What labels tell us now: Selected GI toxicities by class and agent (latest U.S. labels)

Label-based incidences below reflect the most recently posted U.S. prescribing information as of September 1, 2025, and illustrate the range of GI risks that pathologists and oncologists will encounter.

For the multi-kinase VEGFR inhibitor regorafenib, GI toxicity is common across pivotal trials. In colorectal cancer (CORRECT), diarrhea occurred in 43% of regorafenib-treated patients with grade ≥3 in 8%; in hepatocellular carcinoma (RESORCE), diarrhea occurred in 41% with grade ≥3 in 3%. The label also highlights hemorrhage and GI perforation/fistula under Warnings and Precautions [63].

Lenvatinib demonstrates substantial GI toxicity across lines and combinations. In the CLEAR trial (lenvatinib plus pembrolizumab) for renal cell carcinoma, diarrhea affected 62% (grade 3–4, 10%); in hepatocellular carcinoma (REFLECT), diarrhea occurred in 39% (grade 3–4, 4%). Dose modifications for diarrhea are frequent in combination regimens [74].

Cabozantinib’s label quantifies high rates of diarrhea across indications, with 74% (grade 3–4, 11%) in RCC (METEOR) and 54% (grade 3–4, 10%) in HCC (CELESTIAL). Warnings include hemorrhage and a labeled risk of GI perforations/fistulae (~1%) across studies [75].

Among anti-VEGF antibodies and biosimilars, bevacizumab-product labeling (e.g., the 2025 Jobevne [bevacizumab-nwgd] label) states serious GI perforation in 0.3%–3% across clinical studies, with most events within ~50 days of first dose; dose-modification guidance recommends permanent discontinuation for any GI perforation [76].

For ADCs, trastuzumab deruxtecan (fam-trastuzumab deruxtecan-nxki) lists common GI events—nausea, vomiting, constipation, diarrhea—across multiple indications. In DESTINY-Breast01 pooled safety, nausea occurred in 79%, vomiting 47%, diarrhea 29% (grade ≥3 diarrhea 1.7%). In the gastric-cancer trial DESTINY-Gastric01, nausea occurred in 63%, diarrhea 32% (grade ≥3, 2.4%), and vomiting 26%. Although interstitial lung disease dominates boxed warnings, the label documents clinically significant GI adverse reactions, dose interruptions, and reductions attributable to nausea/diarrhea [77].

Sacituzumab govitecan (TROP-2 ADC) carries prominent GI signals on label: across populations, diarrhea occurred in ~64% with grade 3–4 in ~11%; in the randomized ASCENT trial, diarrhea occurred in 59% (grade 3–4, 11%), nausea 57% (grade 3–4, 3%), vomiting 33% (grade 3–4, 2%). The label outlines a stepwise antidiarrheal algorithm and documents rare complications such as intestinal perforation following severe diarrhea [78].

These label-anchored numbers should be interpreted in the context of indication, combination partners, and trial eligibility; nevertheless, they provide practical anchors for counseling, consent, and thresholding pathologic suspicion when reviewing biopsies in treated patients. FDA labels of selected targeted agents and gastrointestinal toxicities are summarized in Table 2.

**Table 2:** Selected targeted agents: Label-reported gastrointestinal toxicities (Latest FDA Labels, 2023–2025)

Drug (Class)	Primary indication(s)	Any-grade diarrhea (%)	Grade ≥3 diarrhea (%)	Other Key GI toxicities (Label)	Label revision (U.S.)
**Regorafenib (TKI, VEGFR/Multi-kinase)**	Metastatic CRC, GIST, HCC	43 (CRC), 41 (HCC)	8 (CRC), 3 (HCC)	Hemorrhage, GI perforation/fistula	2020 [63]
**Lenvatinib (TKI, VEGFR/Multi-kinase)**	HCC, thyroid carcinoma, endometrial ca. (combo), RCC (combo)	39 (HCC), 62 (RCC combo)	4 (HCC), 10 (RCC combo)	GI bleeding, perforation (rare)	2025 [74]
**Cabozantinib (TKI, VEGFR/MET)**	RCC, HCC, thyroid carcinoma	54 (HCC), 74 (RCC)	10 (HCC), 11 (RCC)	GI perforation/fistula (~1%), hemorrhage	2021 [75]
**Bevacizumab (Anti-VEGF mAb)**	CRC, NSCLC, RCC, cervical ca., others	N/A (not primary AE)	N/A	GI perforation (0.3–3%), fistula, hemorrhage	2025 (Jobevne label) [76]
**Trastuzumab deruxtecan (ADC, anti-HER2)**	HER2+ gastric and breast ca.	29–32	1–2	Nausea (63–79%), vomiting (26–47%), mucositis	2025 [77]
**Sacituzumab govitecan (ADC, anti-Trop-2)**	mTNBC, urothelial ca., under study in CRC	59–64	11	Nausea (57%), vomiting (33%), risk of perforation (rare)	2025 [78]

Data derived from pivotal clinical trials and most recent U.S. FDA prescribing information as of September 1, 2025. Reported frequencies may vary by trial population, combination partners, and treatment line. Values are rounded to whole numbers for clarity. Abbreviations: CRC: Colorectal cancer; GIST: Gastrointestinal stromal tumor; HCC: Hepatocellular carcinoma; RCC: Renal cell carcinoma; TKI: Tyrosine kinase inhibitor; VEGFR: Vascular endothelial growth factor receptor; ADC: Antibody–drug conjugate; mAb: Monoclonal antibody; mTNBC: Metastatic triple-negative breast cancer.

### How to use FAERS

For each priority drug class, FAERS queries can be structured by generic/brand name and GI-related MedDRA Preferred Terms (PTs). For anti-VEGF agents, include PTs such as “gastrointestinal perforation,” “intestinal fistula,” and “gastrointestinal hemorrhage.” For ADCs, emphasize “diarrhea,” “colitis,” “stomatitis,” and “abdominal pain.” For TKIs, include “diarrhoea,” “mucositis,” and “ischaemic colitis.” When a disproportionality signal emerges, crosswalk it to label language and trial data to decide whether it represents a known, labeled risk (supporting earlier recognition and biopsy triage) or a potentially emergent pattern that merits caution in clinicopathologic interpretation and consideration for safety reporting [79].

### Practical replication for readers in the U.S.

Investigators and pathology trainees can reproduce our approach by using the FAERS Public Dashboard to filter by product name, time window, reporter type, and PTs, exporting de-duplicated line lists for qualitative pattern review. Parallel extraction from the most recent FDA labels via Drugs@FDA ensures that any FAERS signal is contrasted against authoritative, indication-specific incidence and management guidance. This dual workflow supports reproducible, U.S.-centric safety synthesis aligned with national surveillance priorities [72].

### Interpretation caveats for the manuscript

Spontaneous reports in FAERS cannot be used to estimate incidence or compare products; numerators are incomplete, denominators unknown, and reporting can be stimulated by media or regulatory actions. Labels, in turn, distill adverse reactions observed under protocolized monitoring and may under-capture long-latency or off-indication events seen in general practice. Integrating both sources using labels for rates and FAERS for breadth and recency offers the most defensible, U.S.-relevant picture for pathologists adjudicating GI injury patterns in patients receiving targeted therapies [72].

## CLINICAL MANAGEMENT AND IMPLICATIONS

Recognition and management of gastrointestinal (GI) toxicity in patients receiving targeted cancer therapies require a nuanced, multidisciplinary approach. Unlike cytotoxic chemotherapy, where supportive measures are standardized, toxicities from tyrosine kinase inhibitors (TKIs), antibody–drug conjugates (ADCs), and CAR-T therapies are diverse, often class-specific, and can mimic infectious or immune-mediated diseases. Prompt identification and accurate attribution are essential to prevent morbidity and avoid premature discontinuation of effective anticancer therapy.

### Recognition and monitoring

Baseline assessment should include a detailed gastrointestinal history, nutritional status, and review of concomitant medications such as anticoagulants, antiplatelet agents, or NSAIDs that may exacerbate bleeding risk. During therapy, patients should be counseled to promptly report diarrhea, abdominal pain, hematochezia, or new dyspeptic symptoms. For VEGFR-targeted TKIs such as regorafenib, cabozantinib, and lenvatinib, particular vigilance is needed for ischemic-type colitis and hemorrhage, as these may progress rapidly if unrecognized [80, 81]. For ADCs such as trastuzumab deruxtecan and sacituzumab govitecan, nausea, vomiting, and diarrhea are frequent early-onset toxicities; early initiation of antiemetics and antidiarrheals can mitigate severity [82, 83]. In patients receiving CAR-T therapy, gastrointestinal symptoms appearing in the context of cytokine release syndrome (CRS) should raise suspicion for immune-mediated colitis or enteritis [84].

### Diagnostic decision-making

The decision to pursue endoscopic evaluation with biopsy versus supportive management depends on the clinical scenario. In patients with mild diarrhea and no alarming features, empirical antidiarrheal therapy (e.g., loperamide) may suffice. However, in those with persistent, bloody, or severe diarrhea, colonoscopy with biopsy is warranted to differentiate ischemic colitis, apoptotic enteropathy, and infectious causes. Histopathologic recognition of drug-related injury patterns is crucial, as misinterpretation as inflammatory bowel disease or graft-versus-host disease could lead to unnecessary or harmful immunosuppression [59].

### Therapeutic strategies

Management strategies vary by severity and mechanism. For VEGFR-TKI–related diarrhea, first-line treatment includes loperamide and hydration, with dose interruption or reduction for grade ≥3 events. For ADC-related diarrhea, early institution of scheduled loperamide and, in some cases, octreotide is recommended, with temporary drug interruption for severe cases [68, 83]. For CAR-T–related colitis, systemic corticosteroids and cytokine-directed therapy (e.g., tocilizumab) may be necessary in the setting of concurrent CRS, guided by multidisciplinary teams [85]. In all cases, supportive care with aggressive fluid resuscitation, electrolyte correction, and nutritional support are mainstays.

### Dose modification and drug discontinuation

All FDA-approved prescribing information provides detailed dose-modification algorithms, which should be strictly followed. For example, regorafenib requires dose reduction for grade ≥3 diarrhea or GI hemorrhage; cabozantinib mandates permanent discontinuation for GI perforation or fistula [75]. ADCs such as trastuzumab deruxtecan and sacituzumab govitecan require treatment interruption for grade 3 diarrhea and permanent discontinuation for recurrent grade 4 events [77, 78]. Bevacizumab and biosimilars carry a black box warning mandating discontinuation for any GI perforation [76].

### Multidisciplinary collaboration

Optimal care requires close coordination between oncologists, gastroenterologists, and pathologists. Oncologists must provide detailed treatment histories, including start dates and dose adjustments, while pathologists must highlight histologic patterns consistent with drug toxicity. Early gastroenterology involvement allows timely endoscopic assessment and supportive therapy. Such collaboration not only improves outcomes but also contributes to pharmacovigilance by facilitating accurate adverse event reporting.

**Figure 2:**
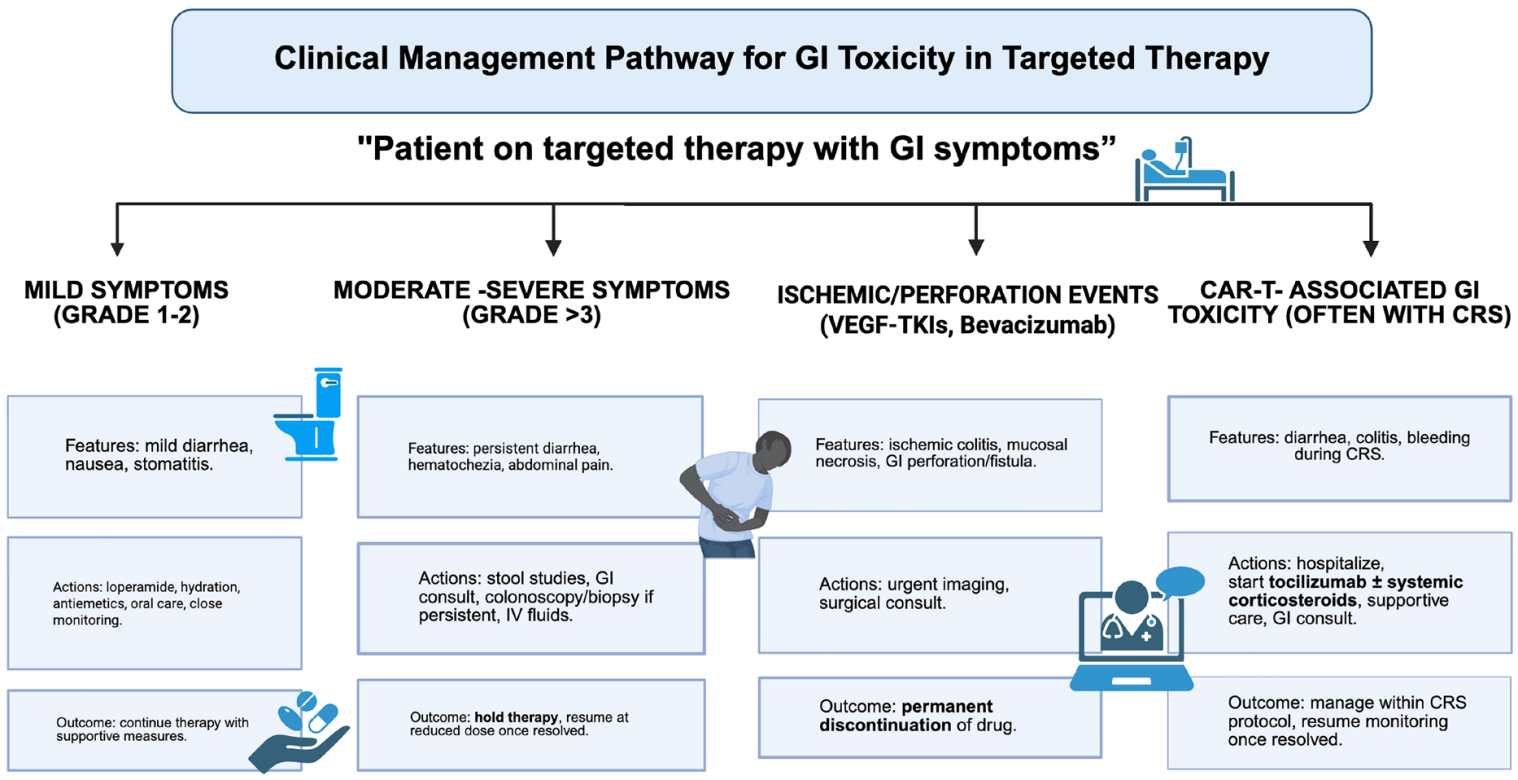
Clinical management pathway for GI toxicity in targeted therapy. This flowchart summarizes management of gastrointestinal adverse events from targeted therapies. Supportive care suffices for mild cases, while moderate to severe symptoms require drug interruption and diagnostic work-up. Ischemic injury or perforation necessitates permanent discontinuation. CAR-T–related GI toxicities, typically occurring in the context of cytokine release syndrome, require systemic immunomodulation with tocilizumab and corticosteroids. Abbreviations: ADC: Antibody–Drug Conjugate; CAR-T: Chimeric Antigen Receptor T-cell; CRS: Cytokine Release Syndrome; CTCAE: Common Terminology Criteria for Adverse Events; GI: Gastrointestinal; TKI: Tyrosine Kinase Inhibitor; VEGF/VEGFR – Vascular Endothelial Growth Factor/(Receptor).

For pathologists, awareness of drug-specific injury patterns can transform biopsy interpretation. An ischemic-type pattern in a regorafenib-treated patient, or apoptotic enteropathy in an everolimus-treated patient, should prompt direct mention of “compatible with targeted-therapy toxicity” in the pathology report, thereby shortening diagnostic timelines and avoiding unnecessary investigations. This patient-safety role is central to pathology’s contribution to modern oncology. Clinical management pathway is illustrated in Figure 2. Practical management of GI toxicities from targeted (non-ICI) therapies are summarized in Table 3.

**Table 3:** Practical management of GI toxicities from targeted (non-ICI) therapies

Drug class (examples)	Common GI adverse events	First-line management (outpatient/initial)	Escalation/when to interrupt, reduce, or stop
**VEGFR/multikinase TKIs (regorafenib, cabozantinib, lenvatinib, sunitinib, sorafenib)**	Diarrhea; abdominal pain; mucosal ulceration; ischemic-type colitis; GI bleeding; (rare) perforation/fistula	Hydration; start loperamide at first loose stool; review/hold aggravating meds (NSAIDs, anticoagulants) when safe; PPI if upper-GI symptoms; stool studies if ≥moderate diarrhea; early GI consult for red flags	Hold drug for persistent grade ≥2 or any bleeding; hospitalize for grade ≥3; colonoscopy if severe/persistent or hematochezia; dose reduce/resume per label when resolved; permanent discontinuation for any GI perforation/fistula; urgent surgical evaluation if suspected
**ADCs (trastuzumab deruxtecan, sacituzumab govitecan)**	Nausea/vomiting, diarrhea, mucositis/stomatitis; colitis (less common)	Prophylactic antiemetics per emetogenic risk; early/scheduled loperamide; oral rehydration and diet modification; oral care for mucositis; rule out infection if ≥moderate	Interrupt for grade 3 diarrhea or persistent grade 2; consider octreotide if refractory; resume at reduced dose per label once ≤grade 1; permanently discontinue for life-threatening/recurrent grade 4 events; manage neutropenia/fever per institutional protocol
**mTOR inhibitor(everolimus)**	Stomatitis/oral mucositis; diarrhea; enteritis (less common)	Topical steroid mouthwash and oral hygiene; analgesia; loperamide PRN; nutrition support	Hold/reduce dose for grade ≥2 mucositis not responding to topical therapy or for grade ≥3 diarrhea; evaluate for candidiasis/HSV if prolonged; resume when ≤grade 1
**PARP inhibitors (olaparib, rucaparib)**	Nausea, vomiting, diarrhea; anorexia	Optimize antiemetics (5-HT3 ± dexamethasone as needed); small frequent meals; loperamide PRN; fluids/electrolytes	Interrupt for grade ≥3 GI AEs; dose reduce/resume per label after recovery; consider switch if recurrent/intolerable
**CAR-T cell therapy (axicabtagene ciloleucel, tisagenlecleucel; solid-tumor constructs in trials)**	Diarrhea/colitis or enteritis, often with CRS; (rare) GI bleeding	If mild and no CRS: supportive care, stool studies; close monitoring	If CRS ≥grade 2 or significant colitis: admit; treat CRS per protocol (tocilizumab ± systemic corticosteroids); GI consult and endoscopy if severe or persistent; exclude infection; nutrition support; escalate immunosuppression per institutional algorithm

This cancer therapies require a nuanced, multidisciplinary summarizes practical, label-concordant steps for initial and escalation management of gastrointestinal adverse events (AEs) from non-ICI targeted therapies. “Grade” refers to CTCAE severity. Always cross-check agent-specific U.S. prescribing information for exact thresholds, dose-modification steps, and contraindications. Infectious workup (including C. difficile) should be considered for ≥moderate diarrhea in all classes. Multidisciplinary coordination (oncology–GI–pathology) is recommended for severe or unclear cases. Abbreviations: ADC: antibody–drug conjugate; AE: adverse event; CAR-T: chimeric antigen receptor T-cell; CRS: cytokine release syndrome; CTCAE: Common Terminology Criteria for Adverse Events; GI: gastrointestinal; ICI: immune checkpoint inhibitor; mTOR: mammalian target of rapamycin; NSAID: nonsteroidal anti-inflammatory drug; PPI: proton-pump inhibitor; TKI: tyrosine kinase inhibitor; VEGF/VEGFR: vascular endothelial growth factor/(receptor).

## NATIONAL POLICY AND PATIENT-SAFETY CONSIDERATIONS

The safe deployment of targeted, non–immune checkpoint cancer therapies in the United States depends on an ecosystem that couples post-marketing pharmacovigilance with standardized toxicity grading, interoperable data capture, and clear communication in the diagnostic record. For pathology and oncology services that routinely encounter gastrointestinal (GI) adverse events from these agents, aligning local practice with national frameworks strengthens patient safety and improves signal detection.

### Pharmacovigilance infrastructure and reporting

At the federal level, two complementary systems underpin drug-safety surveillance. The FDA Adverse Event Reporting System (FAERS) aggregates spontaneous reports from clinicians, manufacturers, and patients and provides a public dashboard for drug- and event-specific queries and data export. FAERS is indispensable for signal detection, although it cannot estimate incidence or prove causality and must be interpreted alongside trial and label data [79]. Clinicians can (and should) submit suspected adverse events directly through FDA MedWatch using the online portal or FDA Form 3500 for health professionals; instructions and form fields explicitly support reporting of clinical findings, diagnostics, and narrative causality assessment [86–88]. Beyond spontaneous reports, the FDA’s Sentinel Initiative conducts active surveillance using large, curated electronic healthcare datasets to evaluate safety questions at scale, enabling more rapid, population-level assessments when signals emerge in FAERS or literature [89].

### Risk evaluation programs and recent regulatory changes

For high-risk products, the FDA may require a Risk Evaluation and Mitigation Strategy (REMS) to ensure benefits outweigh risks and to formalize elements to assure safe use (e.g., site certification, drug-specific monitoring). REMS are imposed on a limited subset of products and are periodically re-evaluated as evidence evolves [90]. In June 2025, the FDA eliminated the REMS requirements for currently approved BCMA- and CD19-directed autologous CAR-T cell therapies after determining that existing labeling and risk-communication tools were sufficient; updated labeling reflects this policy change. This removal reduces administrative burden without changing the expectation for facilities to maintain readiness (e.g., access to tocilizumab, escalation protocols) when treating patients at risk for cytokine release syndrome and neurotoxicity [91]. These developments illustrate how risk-mitigation obligations can change over time and should be monitored by institutions providing advanced cell therapies.

### Standardized toxicity grading and patient-reported outcomes

Uniform grading enables consistent management and cross-study comparability. The National Cancer Institute’s Common Terminology Criteria for Adverse Events (CTCAE) is the U.S. standard for defining and grading adverse events in oncology; the current release is CTCAE v6.0 (2025), which updates terms, grades, and MedDRA mappings used in trials and practice [92]. Because many GI toxicities are symptomatic, integrating patient-reported outcomes improves detection and timeliness. The NCI’s PRO-CTCAE system provides validated items and form builders for capturing patient-reported frequency, severity, and interference of symptomatic adverse events (including diarrhea, abdominal pain, and mucositis), complementing clinician-graded CTCAE and facilitating earlier intervention [93].

### Pathology reporting, terminologies, and data standards

From a safety perspective, pathology reports are critical evidence in adverse-event adjudication. Synoptic and structured reporting—long promoted by the College of American Pathologists (CAP) to ensure completeness and clarity—helps clinicians and pharmacovigilance teams link histopathologic patterns to suspect therapies (e.g., “ischemic-type colitis compatible with VEGF-pathway inhibition”) and shortens time to action [94]. Using standard terminologies strengthens interoperability: the Medical Dictionary for Regulatory Activities (MedDRA) is the international regulatory vocabulary used by FDA and industry for coding adverse events and aligns with CTCAE terms and grades [95]. At the EHR level, adoption of HL7 FHIR-based oncology profiles such as mCODE (Minimal Common Oncology Data Elements) promotes consistent capture of treatment exposures, toxicities, and outcomes across systems, improving the quality of real-world safety data that feed institutional dashboards, registries, and, ultimately, national surveillance [96].

### Practical implications for U.S. centers

Clinically, these national frameworks translate to several operational imperatives. First, institutions should maintain a simple, visible pathway for clinicians and pathology services to submit MedWatch reports when biopsy-proven or strongly suspected targeted-therapy GI injuries occur, ideally pre-populated with medication names, dates, and pathology descriptors. Second, endoscopy and pathology teams should document CTCAE grade (when feasible from clinical data) and use language that supports MedDRA coding. Third, oncology programs offering TKIs, ADCs, or CAR-T should keep living protocols synchronized with label updates and regulatory changes, such as the 2025 CAR-T REMS elimination, so that order sets, checklists, and escalation pathways remain current. Finally, building interoperable data flows (e.g., mCODE-conformant exports) facilitates local safety learning and contributes to national post-marketing knowledge.

## RESEARCH GAPS AND FUTURE DIRECTIONS

Despite the expanding body of evidence on gastrointestinal (GI) toxicities from non–immune checkpoint targeted therapies, several important gaps persist. Addressing these unmet needs will not only improve patient safety but also advance the integration of pathology into national oncology care.

### Limited clinicopathologic correlation

Most clinical trials and FDA labels report rates of GI adverse events based on patient symptoms and laboratory findings but rarely include systematic histopathology data. Consequently, the spectrum of microscopic injury patterns apoptotic enteropathy, ischemic-type changes, and ulcerative colitis-like lesions remains under-characterized [97]. Future multi-institutional registries that link biopsy material with treatment exposure, CTCAE grade, and clinical outcome would strengthen diagnostic accuracy and guide tailored interventions.

### Underreporting in pharmacovigilance systems and integration of patient-reported outcomes and digital health

Spontaneous reporting to FAERS captures only a fraction of actual events. Even biopsy-confirmed drug-related toxicities are often not submitted, creating a blind spot for regulators and industry. Developing automated or semi-automated pipelines that extract structured toxicity data (e.g., MedDRA-coded pathology reports) into pharmacovigilance systems could enhance real-world signal detection [98]. While the NCI’s PRO-CTCAE framework allows structured capture of GI symptoms such as diarrhea, nausea, and abdominal pain, adoption is inconsistent outside clinical trials. Integrating PRO-CTCAE items into routine EHR workflows and linking them with pathology findings could accelerate recognition of evolving toxicities. Mobile health applications with real-time symptom reporting offer another avenue for early intervention and should be tested in populations on high-risk agents like ADCs [99].

### Microbiome and host genetic susceptibility and comparative effectiveness of management strategies

Preclinical and translational studies suggest that the gut microbiome influences toxicity risk from TKIs and immunotherapies, but robust data in ADCs and CAR-T therapies are lacking. Similarly, host genetic polymorphisms in drug-metabolizing enzymes and DNA-repair pathways may modulate GI toxicity [100]. Large-scale, prospective cohorts integrating multi-omics with clinical phenotyping could identify predictive biomarkers of toxicity.

Evidence comparing different management strategies for gastrointestinal toxicity from targeted therapies remains limited, but several themes have emerged. Corticosteroid-based approaches are effective for inflammatory or immune-mediated injury patterns most notably those associated with CAR-T therapies and bispecific antibodies, where cytokine-driven colitis often responds rapidly to systemic steroids and IL-6 pathway blockade [48]. In contrast, GI toxicities from TKIs, VEGF-pathway inhibitors, and ADCs are frequently non-immune in mechanism, and steroid therapy offers little benefit; management instead relies on supportive care, dose interruption, or permanent discontinuation in cases of ischemic injury or perforation risk. Limited comparative data suggest that nonsteroid strategies such as aggressive hydration, antimotility agents, bile acid sequestrants, and octreotide may be more effective in TKI- or ADC-associated diarrhea, whereas steroid exposure may delay mucosal regeneration in apoptosis-predominant injury [101]. However, standardized trials comparing steroid versus nonsteroid approaches are lacking, and current practice is guided largely by mechanistic rationale, small case series, and extrapolation from ICI-related colitis literature. This highlights a significant evidence gap, underscoring the need for prospective, class-specific studies to inform optimal management algorithms.

### Policy and real-world implementation science

The removal of REMS requirements for CAR-T therapies in 2025 illustrates how regulatory frameworks evolve with emerging safety data. However, whether such changes affect real-world toxicity detection or reporting remains unknown. Implementation science approaches should evaluate how institutional workflows, structured reporting, and interoperability standards (e.g., mCODE) influence safety outcomes and national pharmacovigilance [96, 102].

## CONCLUSIONS

Targeted, non–immune checkpoint therapies including tyrosine kinase inhibitors, antibody–drug conjugates, and CAR-T constructs—have fundamentally reshaped gastrointestinal oncology, offering patients unprecedented survival gains across colorectal cancer, gastric and gastroesophageal junction cancer, hepatocellular carcinoma, and gastrointestinal stromal tumors. However, these advances come with a unique spectrum of gastrointestinal toxicities that differ mechanistically and histologically from those associated with cytotoxic chemotherapy or immune checkpoint inhibitors.

**Figure 3:**
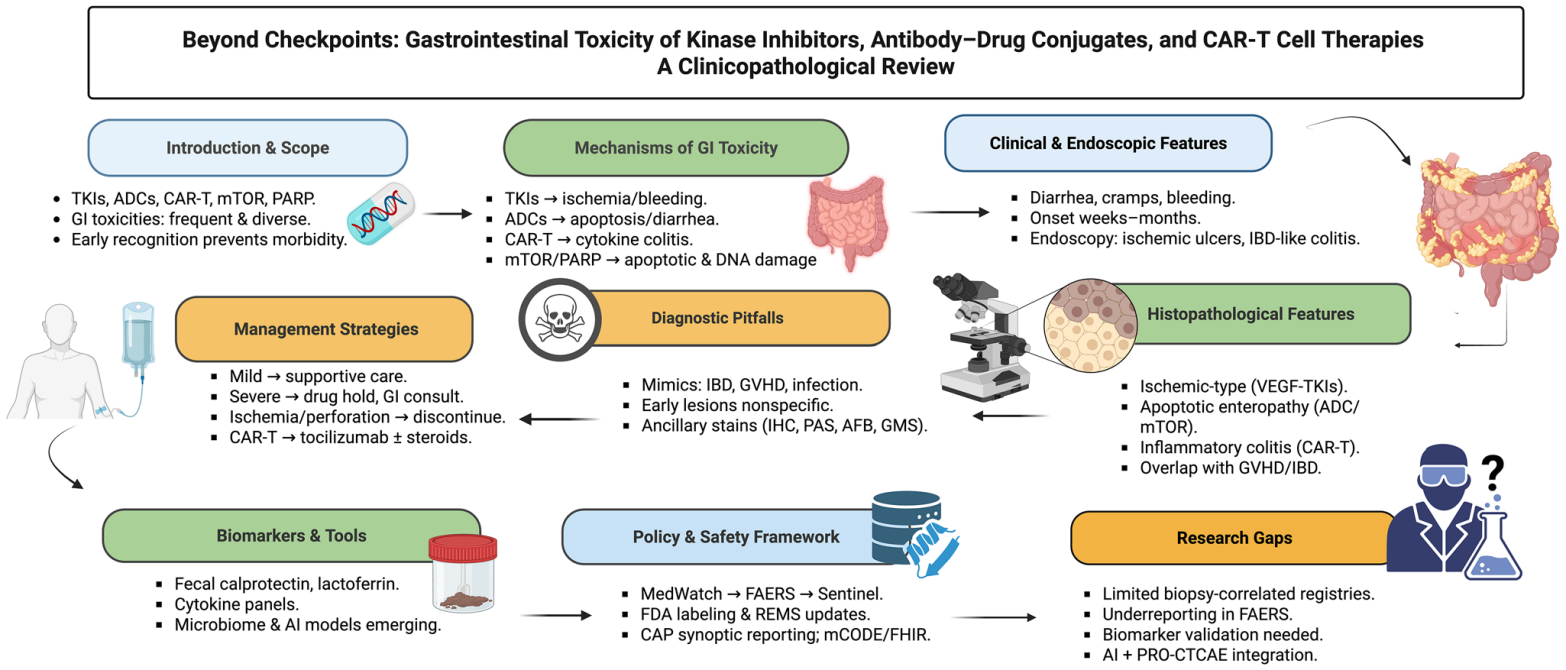
Gastrointestinal toxicity of kinase inhibitors, antibody-drug conjugates, and CAR-T cell therapies. This review highlights how these toxicities are rooted in on-target epithelial injury, anti-angiogenic ischemia, apoptotic enteropathy, and cytokine-driven inflammation. Their manifestations are clinically heterogeneous, spanning diarrhea, mucositis, ischemic colitis, bleeding, and, in rare cases, perforation. Pathologists play a central role in recognizing distinct histopathologic patterns and avoiding misdiagnosis, while oncologists must integrate supportive care, structured dose modification, and multidisciplinary input to optimize outcomes. Abbreviations: ADC: Antibody-Drug Conjugate; AE: Adverse Event; Al: Artificial Intelligence; CAR-T: Chimeric Antigen Receptor T-cell; CAP: College of American Pathologists; CRS: Cytokine Release Syndrome; CTCAE: Common Terminology Criteria for Adverse Events; FAERS: FDA Adverse Event Reporting System; FHIR: Fast Healthcare Interoperability Resources; GI: Gastrointestinal; GIST: Gastrointestinal Stromal Tumor; GVHD: Graft-Versus-Host Disease; HCC: Hepatocellular Carcinoma; IBD: Inflammatory Bowel Disease; ICI: Immune Checkpoint Inhibitor; IHC: lmmunohistochemistry; MedDRA: Medical Dictionary for Regulatory Activities; mCODE: Minimal Common Oncology Data Elements; mTOR: Mammalian Target of Rapamycin; PARP: Poly(ADP-ribose) Polymerase; PAS: Periodic Acid-Schiff; PRO-CTCAE: Patient-Reported Outcomes version of CTCAE; TKI: Tyrosine Kinase Inhibitor; VEGF/VEGFR: Vascular Endothelial Growth Factor/(Receptor).

As seen in Figure 3, this review highlights how these toxicities are rooted in on-target epithelial injury, anti-angiogenic ischemia, apoptotic enteropathy, and cytokine-driven inflammation. Their manifestations are clinically heterogeneous, spanning diarrhea, mucositis, ischemic colitis, bleeding, and, in rare cases, perforation. Pathologists play a central role in recognizing distinct histopathologic patterns and avoiding misdiagnosis, while oncologists must integrate supportive care, structured dose modification, and multidisciplinary input to optimize outcomes.

Beyond clinical management, this field intersects with national safety frameworks, including FAERS pharmacovigilance, CTCAE/PRO-CTCAE standardization, and evolving FDA policies such as REMS updates for CAR-T therapies. The alignment of pathology reporting with MedDRA coding and structured standards such as mCODE strengthens interoperability and supports the U.S. cancer surveillance infrastructure.

Looking forward, progress will depend on bridging critical research gaps particularly in real-world histopathology correlation, microbiome interactions, and predictive biomarkers of toxicity while embedding digital tools for patient-reported outcomes and pharmacovigilance. By integrating pathology, oncology, and regulatory science, the U.S. can enhance patient safety, reduce treatment interruptions, and accelerate the safe adoption of next-generation targeted therapies.

## References

[1] Becker JC, Muller-Tidow C, Serve H, Domschke W, Pohle T. Role of receptor tyrosine kinases in gastric cancer: new targets for a selective therapy. World J Gastroenterol. 2006; 12:3297–305. 10.3748/wjg.v12.i21.3297. 16733844 PMC4087885

[2] Singh D, Dheer D, Samykutty A, Shankar R. Antibody drug conjugates in gastrointestinal cancer: From lab to clinical development. J Control Release. 2021; 340:1–34. 10.1016/j.jconrel.2021.10.006. 34673122

[3] Zhang Q, Song X, Liu J, Zhou X. Prospects of engineered bacteria-assisted CAR T Cell therapy in gastrointestinal cancers. Oncol Rev. 2025; 19:1581856. 10.3389/or.2025.1581856. 40297102 PMC12034723

[4] Botta GP, Chao J, Ma H, Hahn M, Sierra G, Jia J, Hendrix AY, Nolte Fong JV, Ween A, Vu P, Miller A, Choi M, Heyman B, et al. Metastatic gastric cancer target lesion complete response with Claudin18.2-CAR T cells. J Immunother Cancer. 2024; 12:e007927. 10.1136/jitc-2023-007927. 38316518 PMC10860094

[5] Secombe KR, Van Sebille YZA, Mayo BJ, Coller JK, Gibson RJ, Bowen JM. Diarrhea Induced by Small Molecule Tyrosine Kinase Inhibitors Compared With Chemotherapy: Potential Role of the Microbiome. Integr Cancer Ther. 2020; 19:1534735420928493. 10.1177/1534735420928493. 32493068 PMC7273583

[6] Liu C, Amin R, Shatila M, Short N, Altan M, Shah A, Alhalabi O, Okhuysen P, Thomas AS, Wang Y. Clinical characteristics and outcomes of tyrosine kinase inhibitor-related lower GI adverse effects. J Cancer Res Clin Oncol. 2023; 149:3965–76. 10.1007/s00432-022-04316-3. 36030431 PMC11797313

[7] Nguyen TD, Bordeau BM, Balthasar JP. Mechanisms of ADC Toxicity and Strategies to Increase ADC Tolerability. Cancers (Basel). 2023; 15:713. 10.3390/cancers15030713. 36765668 PMC9913659

[8] Cheng Y, Lu J, Zhang C, Yan W, Zhu P, Qin Q, Gong L. Overview of antibody-drug conjugates nonclinical and clinical toxicities and related contributing factors. Antib Ther. 2025; 8:124–44. 10.1093/abt/tbaf004. 40491603 PMC12146482

[9] Li Y, Ming Y, Fu R, Li C, Wu Y, Jiang T, Li Z, Ni R, Li L, Su H, Liu Y. The pathogenesis, diagnosis, prevention, and treatment of CAR-T cell therapy-related adverse reactions. Front Pharmacol. 2022; 13:950923. 10.3389/fphar.2022.950923. 36313336 PMC9616161

[10] Müller A. Rare Toxicities of CAR T-Cell Therapy: Cardiovascular, Hematopoietic and Other Infrequent Adverse Events. healthbook TIMES Onco Hema. 2024; 20:26–33. 10.36000/HBT.OH.2024.20.147.

[11] Brudno JN, Kochenderfer JN. Recent advances in CAR T-cell toxicity: Mechanisms, manifestations and management. Blood Rev. 2019; 34:45–55. 10.1016/j.blre.2018.11.002. 30528964 PMC6628697

[12] Tang X, Wang C, Li Y, Tang J, Zhang G, Chen L. Signal detection and safety analysis of three tyrosine kinase inhibitors for HER-2 positive breast cancer: a retrospective study based on the FAERS database. Front Pharmacol. 2025; 16:1538881. 10.3389/fphar.2025.1538881. 40129939 PMC11931018

[13] Fan Y, Wu T, Xu P, Yang C, An J, Zhang H, Abbas M, Dong X. Neratinib safety evaluation: real-world adverse event analysis from the FAERS database. Front Pharmacol. 2024; 15:1425171. 10.3389/fphar.2024.1425171. 39346561 PMC11427278

[14] Han J, Zhai X, Tao X, Li Y, Zhao Z, Yu Z, Dong D, Yang S, Lv L. Pharmacovigilance study of adverse reactions of anti-HER-2 drugs for the treatment of HER-2-positive breast cancer based on the FAERS database. Breast Cancer Res. 2025; 27:54. 10.1186/s13058-025-02013-w. 40205546 PMC11983758

[15] Tan AD, Willemsma K, MacNeill A, DeVries K, Srikanthan A, McGahan C, Hamilton T, Li H, Blanke CD, Simmons CE. Tyrosine kinase inhibitors significantly improved survival outcomes in patients with metastatic gastrointestinal stromal tumour: a multi-institutional cohort study. Curr Oncol. 2020; 27:e276–82. 10.3747/co.27.5869. 32669934 PMC7339857

[16] Barnett CM, Heinrich MC. Management of tyrosine kinase inhibitor-resistant gastrointestinal stromal tumors. Am Soc Clin Oncol Educ Book. 2012; 663–68. 10.14694/EdBook_AM.2012.32.66. 24451815

[17] He C, Wang Z, Yu J, Mao S, Xiang X. Current Drug Resistance Mechanisms and Treatment Options in Gastrointestinal Stromal Tumors: Summary and Update. Curr Treat Options Oncol. 2024; 25:1390–05. 10.1007/s11864-024-01272-7. 39441520 PMC11541409

[18] Liegl B, Kepten I, Le C, Zhu M, Demetri GD, Heinrich MC, Fletcher CD, Corless CL, Fletcher JA. Heterogeneity of kinase inhibitor resistance mechanisms in GIST. J Pathol. 2008; 216:64–74. 10.1002/path.2382. 18623623 PMC2693040

[19] da Fonseca LG, Reig M, Bruix J. Tyrosine Kinase Inhibitors and Hepatocellular Carcinoma. Clin Liver Dis. 2020; 24:719–37. 10.1016/j.cld.2020.07.012. 33012455

[20] Mou L, Tian X, Zhou B, Zhan Y, Chen J, Lu Y, Deng J, Deng Y, Wu Z, Li Q, Song Y, Zhang H, Chen J, et al. Improving Outcomes of Tyrosine Kinase Inhibitors in Hepatocellular Carcinoma: New Data and Ongoing Trials. Front Oncol. 2021; 11:752725. 10.3389/fonc.2021.752725. 34707994 PMC8543014

[21] Ryan AJ, Wedge SR. ZD6474--a novel inhibitor of VEGFR and EGFR tyrosine kinase activity. Br J Cancer. 2005 (Suppl 1); 92:S6–13. 10.1038/sj.bjc.6602603. 15928657 PMC2362058

[22] Saleh K, Khoury R, Khalife N, Chahine C, Ibrahim R, Tikriti Z, Le Cesne A. Mechanisms of action and resistance to anti-HER2 antibody-drug conjugates in breast cancer. Cancer Drug Resist. 2024; 7:22. 10.20517/cdr.2024.06. 39050884 PMC11267152

[23] Kawakami H, Nakanishi K, Makiyama A, Konishi H, Morita S, Narita Y, Sugimoto N, Minashi K, Imano M, Inamoto R, Kodera Y, Kume H, Yamaguchi K, et al, and EN-DEAVOR Study Group. Real-world effectiveness and safety of trastuzumab-deruxtecan in Japanese patients with HER2-positive advanced gastric cancer (EN-DEAVOR study). Gastric Cancer. 2025; 28:51–61. 10.1007/s10120-024-01555-w. 39387986 PMC11706843

[24] Meric-Bernstam F, Makker V, Oaknin A, Oh DY, Banerjee S, González-Martín A, Jung KH, Ługowska I, Manso L, Manzano A, Melichar B, Siena S, Stroyakovskiy D, et al. Efficacy and Safety of Trastuzumab Deruxtecan in Patients With HER2-Expressing Solid Tumors: Primary Results From the DESTINY-PanTumor02 Phase II Trial. J Clin Oncol. 2024; 42:47–58. 10.1200/JCO.23.02005. 37870536 PMC10730032

[25] Pedersini R, Buffoni M, Petrelli F, Ghidini A, di Mauro P, Amoroso V, Parati MC, Laini L, Cosentini D, Schivardi G, Ippolito G, Berruti A, Laganà M. Gastrointestinal Toxicity of Antibody Drug Conjugates (ADCs) in Metastatic Breast Cancer: A Pooled Analysis. Clin Breast Cancer. 2024; 24:411–20. 10.1016/j.clbc.2024.04.003. 38734491

[26] Köhler BC, Haag GM, Le Cornet L, Hoffmeister-Wittmann P, Schmidt M, Manjunath A, Vaquero-Siguero N, Jenzer M, Gimmel M, Stahler A, Stein A, Reichert M, Kasper S, et al. TROPHIT1—a randomized, open-label, multicenter, phase II/III trial of sacituzumab govitecan compared to standard of care in metastatic colorectal cancer patients. ESMO Gastrointest Oncol. 2025; 7:100118. 10.1016/j.esmogo.2024.100118. 41646480 PMC12836553

[27] Diab M. Sacituzumab Govitecan in Combination With Capecitabine for Advanced Gastrointestinal Cancers After Progression on Standard Therapy. clinicaltrials.gov. 2024. https://clinicaltrials.gov/study/NCT06065371.

[28] Abken H. CAR T cell therapies in gastrointestinal cancers: current clinical trials and strategies to overcome challenges. Nat Rev Gastroenterol Hepatol. 2025; 22:463–80. 10.1038/s41575-025-01062-y. 40229574

[29] Chen X, Li P, Tian B, Kang X. Serious adverse events and coping strategies of CAR-T cells in the treatment of malignant tumors. Front Immunol. 2022; 13:1079181. 10.3389/fimmu.2022.1079181. 36569917 PMC9772271

[30] Fortuna GG, Banerjee R, Savid-Frontera C, Song J, Morán-Segura CM, Nguyen JV, Lekakis L, Fernandez-Pol S, Samraj AN, Naresh KN, Vazquez-Martinez M, Baz RC, Spiegel JY, et al. Immune effector cell-associated enterocolitis following chimeric antigen receptor T-cell therapy in multiple myeloma. Blood Cancer J. 2024; 14:180. 10.1038/s41408-024-01167-8. 39414769 PMC11484697

[31] Moreau P, Garfall AL, van de Donk NWC, Nahi H, San-Miguel JF, Oriol A, Nooka AK, Martin T, Rosinol L, Chari A, Karlin L, Benboubker L, Mateos MV, et al. Teclistamab in Relapsed or Refractory Multiple Myeloma. N Engl J Med. 2022; 387:495–505. 10.1056/NEJMoa2203478. 35661166 PMC10587778

[32] Herrera M, Pretelli G, Desai J, Garralda E, Siu LL, Steiner TM, Au L. Bispecific antibodies: advancing precision oncology. Trends Cancer. 2024; 10:893–19. 10.1016/j.trecan.2024.07.002. 39214782

[33] Harter MF, Recaldin T, Gerard R, Avignon B, Bollen Y, Esposito C, Guja-Jarosz K, Kromer K, Filip A, Aubert J, Schneider A, Bacac M, Bscheider M, et al. Analysis of off-tumour toxicities of T-cell-engaging bispecific antibodies via donor-matched intestinal organoids and tumouroids. Nat Biomed Eng. 2024; 8:345–60. 10.1038/s41551-023-01156-5. 38114742 PMC11087266

[34] Malley CO, Pidgeon GP. The mTOR pathway in obesity driven gastrointestinal cancers: Potential targets and clinical trials. BBA Clin. 2015; 5:29–40. 10.1016/j.bbacli.2015.11.003. 27051587 PMC4802403

[35] Feng Y, Chen Y, Ren Y, Cao S, Zhang H. Advancement on Clinical Application of mTOR Inhibitors in Gastrointestinal Cancers. J Biosci Med. 2020; 8:45–57. 10.4236/jbm.2020.84004.

[36] Alhusaini A, Cannon A, Maher SG, Reynolds JV, Lynam-Lennon N. Therapeutic Potential of PARP Inhibitors in the Treatment of Gastrointestinal Cancers. Biomedicines. 2021; 9:1024. 10.3390/biomedicines9081024. 34440228 PMC8392860

[37] Chen H, Hu Y, Zhuang Z, Wang D, Ye Z, Jing J, Cheng X. Advancements and Obstacles of PARP Inhibitors in Gastric Cancer. Cancers (Basel). 2023; 15:5114. 10.3390/cancers15215114. 37958290 PMC10647262

[38] Dewaele L, Fernandes RA. Bispecific T-cell engagers for the recruitment of T cells in solid tumors: a literature review. Immunother Adv. 2025; 5:ltae005. 10.1093/immadv/ltae005. 40083373 PMC11904783

[39] Liu Y, Meng J, Wang G. Risk of selected gastrointestinal toxicities associated with poly (ADP-ribose) polymerase (PARP) inhibitors in the treatment of ovarian cancer: a meta-analysis of published trials. Drug Des Devel Ther. 2018; 12:3013–19. 10.2147/DDDT.S164553. 30271116 PMC6147204

[40] Wang Z, Qu YJ, Cui M. Modulation of stem cell fate in intestinal homeostasis, injury and repair. World J Stem Cells. 2023; 15:354–68. 10.4252/wjsc.v15.i5.354. 37342221 PMC10277971

[41] Klingler S, Hsu KS, Hua G, Martin ML, Adileh M, Baslan T, Zhang Z, Paty PB, Fuks Z, Brown AM, Kolesnick R. Disruption of the crypt niche promotes outgrowth of mutated colorectal tumor stem cells. JCI Insight. 2022; 7:e153793. 10.1172/jci.insight.153793. 35260534 PMC8983138

[42] Choi J, Augenlicht LH. Intestinal stem cells: guardians of homeostasis in health and aging amid environmental challenges. Exp Mol Med. 2024; 56:495–500. 10.1038/s12276-024-01179-1. 38424189 PMC10985084

[43] Walraven M, Witteveen PO, Lolkema MP, van Hillegersberg R, Voest EE, Verheul HM. Antiangiogenic tyrosine kinase inhibition related gastrointestinal perforations: a case report and literature review. Angiogenesis. 2011; 14:135–41. 10.1007/s10456-010-9197-6. 21188500 PMC3102838

[44] Wang X, Gao F, Guan J, Zhang L, Du L, Zhao Y, Gao F, Zhao K, He W, Lin J. mTOR blockade mitigates chemotherapy drug-induced intestinal toxicity via inhibition of pyroptosis. Biochim Biophys Acta Mol Basis Dis. 2025; 1871:167913. 10.1016/j.bbadis.2025.167913. 40398827

[45] Li W, Huang Y, Zhou X, Cheng B, Wang H, Wang Y. CAR-T therapy for gastrointestinal cancers: current status, challenges, and future directions. Braz J Med Biol Res. 2024; 57:e13640. 10.1590/1414-431X2024e13640. 39417449 PMC11484376

[46] Sievers S, Watson G, Johncy S, Adkins S. Recognizing and Grading CAR T-Cell Toxicities: An Advanced Practitioner Perspective. Front Oncol. 2020; 10:885. 10.3389/fonc.2020.00885. 32670871 PMC7327099

[47] Su Z, Lu L, Chen F, Chen J, Chen X. Gut Microbiota and Sunitinib-Induced Diarrhea in Metastatic Renal Cell Carcinoma: A Pilot Study. Cancer Manag Res. 2021; 13:8663–72. 10.2147/CMAR.S328451. 34849023 PMC8612664

[48] He J, Chen Y, Zhao H, Li Y. The interplay between gut bacteria and targeted therapies: implications for future cancer treatments. Mol Med. 2025; 31:58. 10.1186/s10020-025-01108-6. 39948481 PMC11827328

[49] Mohanavelu P, Mutnick M, Mehra N, White B, Kudrimoti S, Hernandez Kluesner K, Chen X, Nguyen T, Horlander E, Thenot H, Kota V, Mitchell CS. Meta-Analysis of Gastrointestinal Adverse Events from Tyrosine Kinase Inhibitors for Chronic Myeloid Leukemia. Cancers (Basel). 2021; 13:1643. 10.3390/cancers13071643. 33915952 PMC8037766

[50] D’Arienzo A, Verrazzo A, Pagliuca M, Napolitano F, Parola S, Viggiani M, Caputo R, Puglisi F, Giuliano M, Del Mastro L, Arpino G, De Laurentiis M, Montemurro F. Toxicity profile of antibody-drug conjugates in breast cancer: practical considerations. EClinicalMedicine. 2023; 62:102113. 10.1016/j.eclinm.2023.102113. 37554126 PMC10404866

[51] Kamba T, McDonald DM. Mechanisms of adverse effects of anti-VEGF therapy for cancer. Br J Cancer. 2007; 96:1788–95. 10.1038/sj.bjc.6603813. 17519900 PMC2359962

[52] Li XF, Tan YN, Cao Y, Xu JH, Zheng S, Yuan Y. A Case Report of Gastrointestinal Hemorrhage and Perforation During Apatinib Treatment of Gastric Cancer. Medicine (Baltimore). 2015; 94:e1661. 10.1097/MD.0000000000001661. 26426663 PMC4616867

[53] Li Z, Wang Z, Shen B, Chen C, Ding X, Song H. Effects of aspirin on the gastrointestinal tract: Pros vs. cons. Oncol Lett. 2020; 20:2567–78. 10.3892/ol.2020.11817. 32782574 PMC7400979

[54] Barney BM, Markovic S, Laack NN, Miller RC, Sarkaria JN, Macdonald OK, Olivier KR. Increased bowel toxicity in patients treated with a vascular endothelial growth factor inhibitor (VEGF-i) following stereotactic body radiotherapy (SBRT). J Clin Oncol. 2012; 30:e14507. 10.1200/jco.2012.30.15_suppl.e14507. 23920388

[55] Jain A, Barge A, Parris CN. Combination strategies with PARP inhibitors in BRCA-mutated triple-negative breast cancer: overcoming resistance mechanisms. Oncogene. 2025; 44:193–07. 10.1038/s41388-024-03227-6. 39572842 PMC11746151

[56] Zhou T, Zhang J. Therapeutic advances and application of PARP inhibitors in breast cancer. Transl Oncol. 2025; 57:102410. 10.1016/j.tranon.2025.102410. 40359851 PMC12142329

[57] Xu Y, Ou J, Zhang C, Chen J, Chen J, Li A, Huang B, Zhao X. Rapamycin promotes the intestinal barrier repair in ulcerative colitis via the mTOR/PBLD/AMOT signaling pathway. Biochim Biophys Acta Mol Basis Dis. 2024; 1870:167287. 10.1016/j.bbadis.2024.167287. 38862095

[58] Pérez-García JM, Gion M, Ruiz-Borrego M, Blancas I, López-Miranda E, Blanch S, Recalde S, Rendo CR, González X, Ancizar N, Morales S, Cortez P, Piwowarska Z, et al. Prevention of sacituzumab govitecan-related neutropenia and diarrhea in patients with HER2-negative advanced breast cancer (PRIMED): an open-label, single-arm, phase 2 trial. EClinicalMedicine. 2025; 85:103309. 10.1016/j.eclinm.2025.103309. 40606525 PMC12221278

[59] Karimi SS, Larman TC, Jain T, Voltaggio L, Birkness-Gartman JE. Chimeric antigen receptor (CAR) T-cell therapy-related gastrointestinal toxicity: Histologic features and morphologic mimics. Hum Pathol. 2025; 158:105791. 10.1016/j.humpath.2025.105791. 40349988

[60] Hassan Z, Schneeweis C, Wirth M, Veltkamp C, Dantes Z, Feuerecker B, Ceyhan GO, Knauer SK, Weichert W, Schmid RM, Stauber R, Arlt A, Krämer OH, et al. MTOR inhibitor-based combination therapies for pancreatic cancer. Br J Cancer. 2018; 118:366–77. 10.1038/bjc.2017.421. 29384525 PMC5808033

[61] Shyam Sunder S, Sharma UC, Pokharel S. Adverse effects of tyrosine kinase inhibitors in cancer therapy: pathophysiology, mechanisms and clinical management. Signal Transduct Target Ther. 2023; 8:262. 10.1038/s41392-023-01469-6. 37414756 PMC10326056

[62] Paplomata E, Zelnak A, O’Regan R. Everolimus: side effect profile and management of toxicities in breast cancer. Breast Cancer Res Treat. 2013; 140:453–62. 10.1007/s10549-013-2630-y. 23907751

[63] Stivagra, highlights of prescribing information. https://www.accessdata.fda.gov/drugsatfda_docs/label/2020/203085s013lbl.pdf.

[64] LiverTox: Clinical and Research Information on Drug-Induced Liver Injury. Bethesda (MD): National Institute of Diabetes and Digestive and Kidney Diseases; 2012. 31643176

[65] Agrawal V, Pourhassan H, Tsai NC, Ngo D, Koller P, Malki MMA, Salhotra A, Ali H, Aribi A, Sandhu KS, Arslan S, Ball B, Otoukesh S, et al. Post-Transplantation Sinusoidal Obstruction Syndrome in Adult Patients with B Cell Acute Lymphoblastic Leukemia Treated with Pretransplantation Inotuzumab. Transplant Cell Ther. 2023; 29:314–20. 10.1016/j.jtct.2023.01.017. 36682470

[66] Senapati J, Jabbour E, Short NJ, Jain N, Haddad F, Bathala T, Kovalenko I, Bidikian A, Ravandi F, Khouri I, Kadia TM, Garris R, Montalban Bravo G, et al. Liver elastography for risk-assessment of liver toxicity and risk factors for Sinusoidal obstruction syndrome in patients with acute lymphoblastic leukemia receiving inotuzumab ozogamicin. Blood Cancer J. 2024; 14:129. 10.1038/s41408-024-01098-4. 39112504 PMC11306742

[67] Wu X, Kilpatrick T, Chau I. Antibody drug conjugate development in gastrointestinal cancers: hopes and hurdles from clinical trials. Cancer Drug Resist. 2018; 1:204–18. 10.20517/cdr.2018.16.

[68] Santorsola M, Capuozzo M, Nasti G, Sabbatino F, Di Mauro A, Di Mauro G, Vanni G, Maiolino P, Correra M, Granata V, Gualillo O, Berretta M, Ottaiano A. Exploring the Spectrum of VEGF Inhibitors’ Toxicities from Systemic to Intra-Vitreal Usage in Medical Practice. Cancers (Basel). 2024; 16:350. 10.3390/cancers16020350. 38254839 PMC10813960

[69] Tang T, Abu-Sbeih H, Ma W, Lu Y, Luo W, Foo WC, Richards DM, Halperin DM, Ge PS, Wang Y. Gastrointestinal Injury Related to Antiangiogenesis Cancer Therapy. Clin Colorectal Cancer. 2020; 19:e117–23. 10.1016/j.clcc.2020.03.002. 32284253

[70] Bomfim I, Saxena R, de Mello ES. Chemotherapy induced colitis. PathologyOutlines.com website. https://www.pathologyoutlines.com/topic/colonchemoinducedcolitis.html.

[71] Zundler S, Vitali F, Kharboutli S, Völkl S, Polifka I, Mackensen A, Atreya R, Neurath MF, Mougiakakos D. Case Report: IBD-like colitis following CAR T cell therapy for diffuse large B cell lymphoma. Front Oncol. 2023; 13:1149450. 10.3389/fonc.2023.1149450. 37284193 PMC10240064

[72] FDA’s Adverse Event Reporting System (FAERS). https://www.fda.gov/drugs/surveillance/fdas-adverse-event-reporting-system-faers.

[73] Shi Y, Yao K, Zhao J, Yue Y, Wu H. Gastrointestinal toxicity of antibody-drug conjugates: a pharmacovigilance study using the FAERS database. BMC Pharmacol Toxicol. 2025; 26:50. 10.1186/s40360-025-00877-4. 40033454 PMC11874441

[74] LENVIMA, Highlights of Prescribing Information. https://www.accessdata.fda.gov/drugsatfda_docs/label/2025/206947s033lbl.pdf.

[75] CABOZANTINIB, Highlights of Prescribing Information. https://www.accessdata.fda.gov/drugsatfda_docs/label/2021/208692s010lbl.pdf.

[76] BEVACIZUMAB-NWGD, Highlights of Prescribing Information. https://www.accessdata.fda.gov/drugsatfda_docs/label/2025/761175s000lbl.pdf.

[77] ENHERTU® Highlights of Prescribing Information. https://www.accessdata.fda.gov/drugsatfda_docs/label/2025/761139s032s035lbl.pdf.

[78] TRODELVY® Highlights of Prescribing Information. https://www.accessdata.fda.gov/drugsatfda_docs/label/2025/761115s059lbl.pdf.

[79] Potter E, Reyes M, Naples J, Dal Pan G. FDA Adverse Event Reporting System (FAERS) Essentials: A Guide to Understanding, Applying, and Interpreting Adverse Event Data Reported to FAERS. Clin Pharmacol Ther. 2025; 118:567–82. 10.1002/cpt.3701. 40384638 PMC12393772

[80] Chhatrala R, Thanavala Y, Iyer R. Targeted therapy in gastrointestinal malignancies. J Carcinog. 2014; 13:4. 10.4103/1477-3163.127639. 24737952 PMC3986534

[81] Fischer-Cartlidge EA. Assessment and management of gastrointestinal toxicities and lab abnormalities related to targeted therapy. Semin Oncol Nurs. 2014; 30:183–9. 10.1016/j.soncn.2014.05.006. 25085030

[82] Farhat J, Sakai H, Tsurutani J. Management of nausea and vomiting induced by antibody-drug conjugates. Breast Cancer. 2025; 32:278–85. 10.1007/s12282-025-01670-1. 39878905 PMC11842424

[83] Kang S, Kim SB. Toxicities and management strategies of emerging antibody-drug conjugates in breast cancer. Ther Adv Med Oncol. 2025; 17:17588359251324889. 10.1177/17588359251324889. 40151551 PMC11946287

[84] Hu Y, Li J, Ni F, Yang Z, Gui X, Bao Z, Zhao H, Wei G, Wang Y, Zhang M, Hong R, Wang L, Wu W, et al. CAR-T cell therapy-related cytokine release syndrome and therapeutic response is modulated by the gut microbiome in hematologic malignancies. Nat Commun. 2022; 13:5313. 10.1038/s41467-022-32960-3. 36085303 PMC9461447

[85] Ye K, Yu C, Shen Z. Severe refractory colitis after intraperitoneal infusion of CEA-directed CAR T cells in patients with colorectal cancer. Ther Adv Med Oncol. 2024; 16:17588359241309825. 10.1177/17588359241309825. 39734708 PMC11672468

[86] Craigle V. MedWatch: The FDA Safety Information and Adverse Event Reporting Program. J Med Libr Assoc. 2007; 95:224–25. 10.3163/1536-5050.95.2.224.

[87] MedWatch Forms for FDA Safety Reporting. https://www.fda.gov/safety/medical-product-safety-information/medwatch-forms-fda-safety-reporting.

[88] Instructions for Completing Form FDA 3500. https://www.fda.gov/safety/medwatch-forms-fda-safety-reporting/instructions-completing-form-fda-3500.

[89] Brown JS, Mendelsohn AB, Nam YH, Maro JC, Cocoros NM, Rodriguez-Watson C, Lockhart CM, Platt R, Ball R, Dal Pan GJ, Toh S. The US Food and Drug Administration Sentinel System: a national resource for a learning health system. J Am Med Inform Assoc. 2022; 29:2191–200. 10.1093/jamia/ocac153. 36094070 PMC9667154

[90] Risk Evaluation and Mitigation Strategies | REMS. https://www.fda.gov/drugs/drug-safety-and-availability/risk-evaluation-and-mitigation-strategies-rems.

[91] Advocacy Alert: FDA Eliminate REMS for BCMA-and CD19-Directed Autologous CAR T Cell Immunotherapies. https://www.astct.org/Nucleus/Article/advocacy-alert-fda-eliminate-rems-for-bcma-and-cd19-directed-autologous-car-t-cell-immunotherapies.

[92] Basch E, Reeve BB, Mitchell SA, Clauser SB, Minasian LM, Dueck AC, Mendoza TR, Hay J, Atkinson TM, Abernethy AP, Bruner DW, Cleeland CS, Sloan JA, et al. Development of the National Cancer Institute’s patient-reported outcomes version of the common terminology criteria for adverse events (PRO-CTCAE). J Natl Cancer Inst. 2014; 106:dju244. 10.1093/jnci/dju244. 25265940 PMC4200059

[93] The PRO-CTCAE Measurement System. https://healthcaredelivery.cancer.gov/pro-ctcae/measurement.html.

[94] CTCAE and AE Reporting. https://dctd.cancer.gov/research/ctep-trials/for-sites/adverse-events.

[95] Setser A. Use of MedDRA® in CTCAE and in the Biopharmaceutical Industry. https://admin.meddra.org/sites/default/files/page/documents_insert/nci_alliance_ann_setser_2012_0.pdf.

[96] Osterman TJ, Terry M, Miller RS. Improving Cancer Data Interoperability: The Promise of the Minimal Common Oncology Data Elements (mCODE) Initiative. JCO Clin Cancer Inform. 2020; 4:993–1001. 10.1200/CCI.20.00059. 33136433 PMC7713551

[97] Zhang ML, Deshpande V. Histopathology of Gastrointestinal Immune-related Adverse Events: A Practical Review for the Practicing Pathologist. Am J Surg Pathol. 2022; 46:e15–26. 10.1097/PAS.0000000000001730. 33999556

[98] Schilder JM, Golembesky A, Boyle TAC, Ye GL, Kuplast J. Commentary: Adverse event profiles of PARP inhibitors: analysis of spontaneous reports submitted to FAERS. Front Pharmacol. 2023; 14:1241524. 10.3389/fphar.2023.1241524. 37663271 PMC10468970

[99] Basch E, Becker C, Rogak LJ, Schrag D, Reeve BB, Spears P, Smith ML, Gounder MM, Mahoney MR, Schwartz GK, Bennett AV, Mendoza TR, Cleeland CS, et al. Composite grading algorithm for the National Cancer Institute’s Patient-Reported Outcomes version of the Common Terminology Criteria for Adverse Events (PRO-CTCAE). Clin Trials. 2021; 18:104–14. 10.1177/1740774520975120. 33258687 PMC7878323

[100] Wang Y, Jenq RR, Wargo JA, Watowich SS. Microbiome influencers of checkpoint blockade-associated toxicity. J Exp Med. 2023; 220:e20220948. 10.1084/jem.20220948. 36622383 PMC9836236

[101] Sun K, Wang X, Zhang H, Lin G, Jiang R. Management and Mechanisms of Diarrhea Induced by Tyrosine Kinase Inhibitors in Human Epidermal Growth Factor Receptor-2-Positive Breast Cancer. Cancer Control. 2024; 31:10732748241278039. 10.1177/10732748241278039. 39159918 PMC11334140

[102] FDA Eliminates Risk Evaluation and Mitigation Strategies (REMS) for Autologous Chimeric Antigen Receptor CAR T cell Immunotherapies. https://www.fda.gov/news-events/press-announcements/fda-eliminates-risk-evaluation-and-mitigation-strategies-rems-autologous-chimeric-antigen-receptor.

